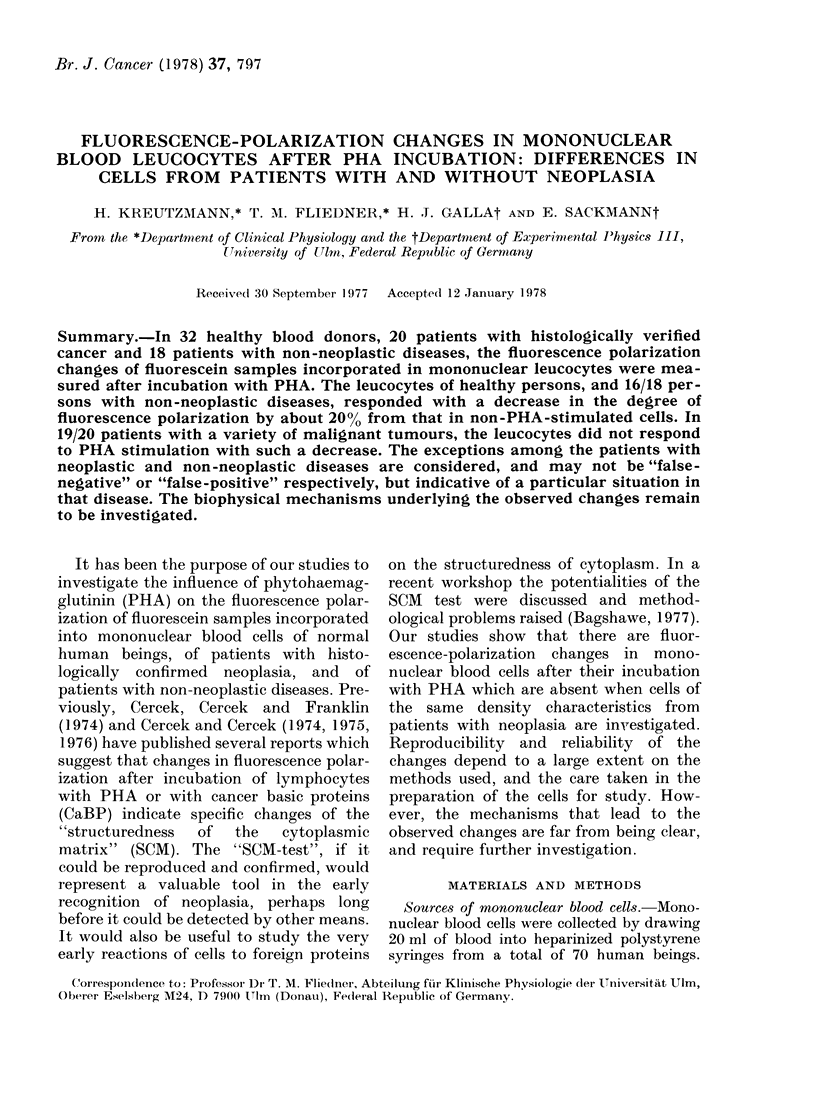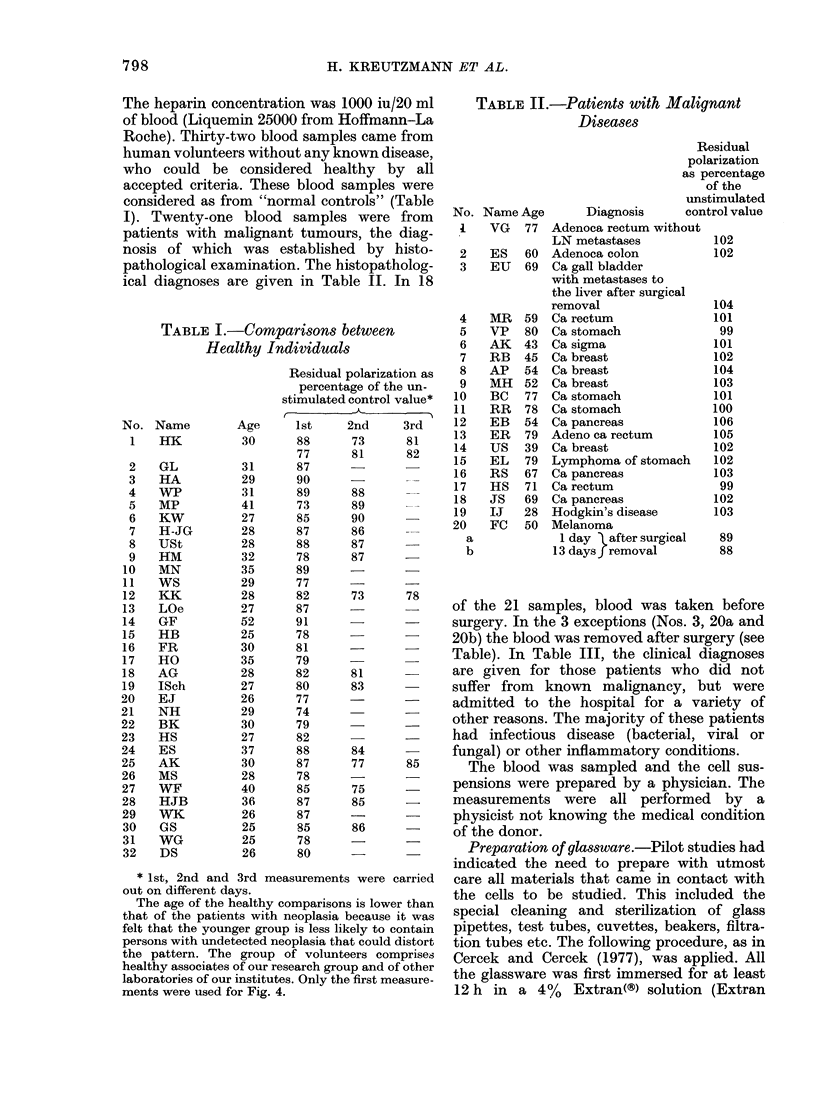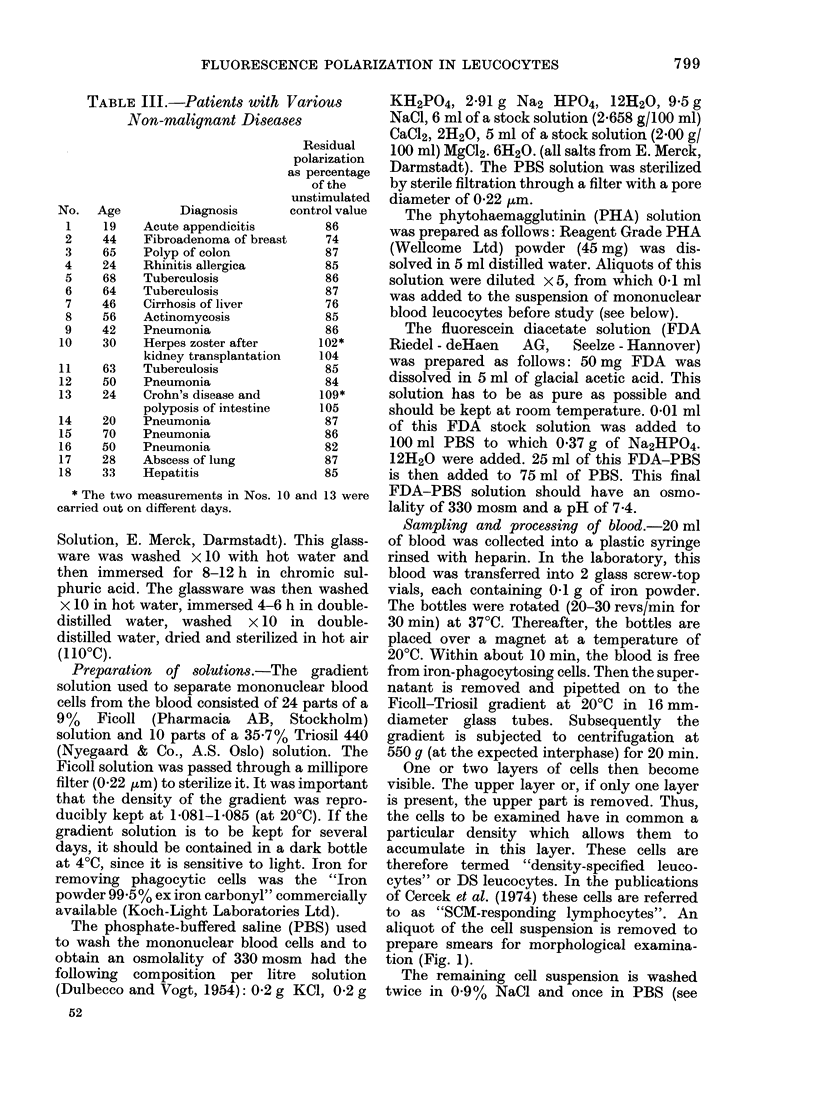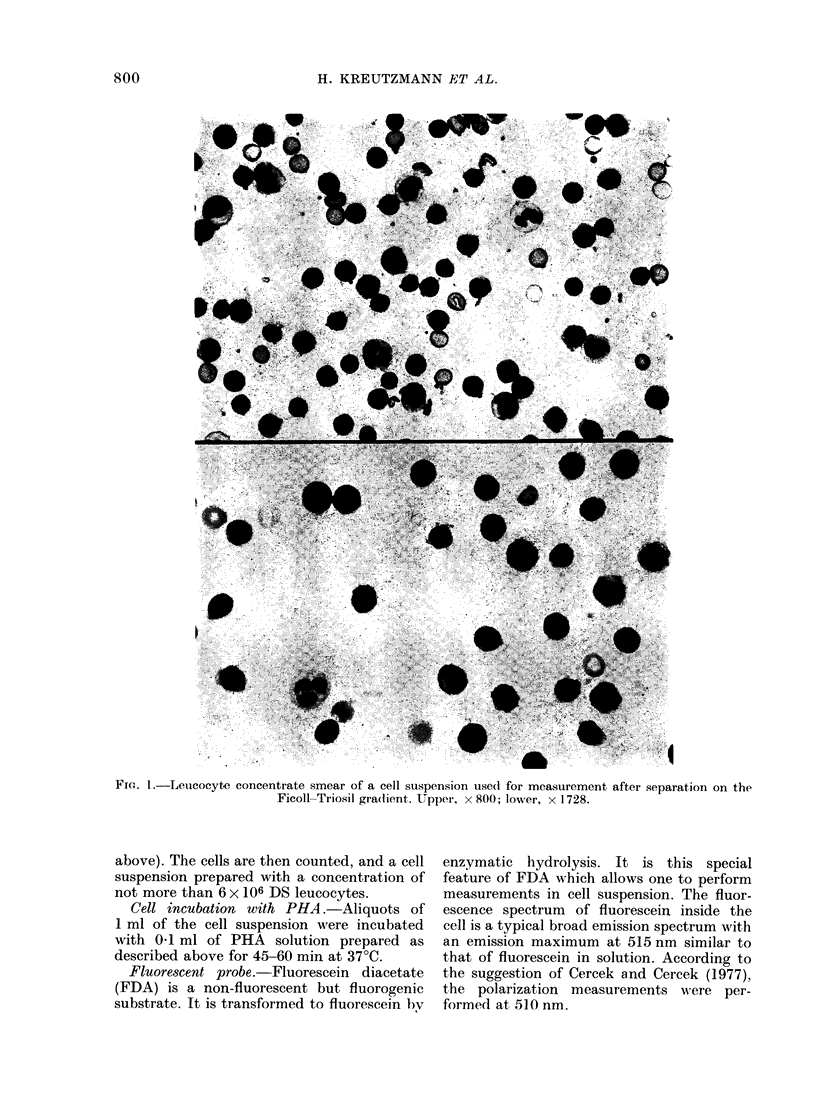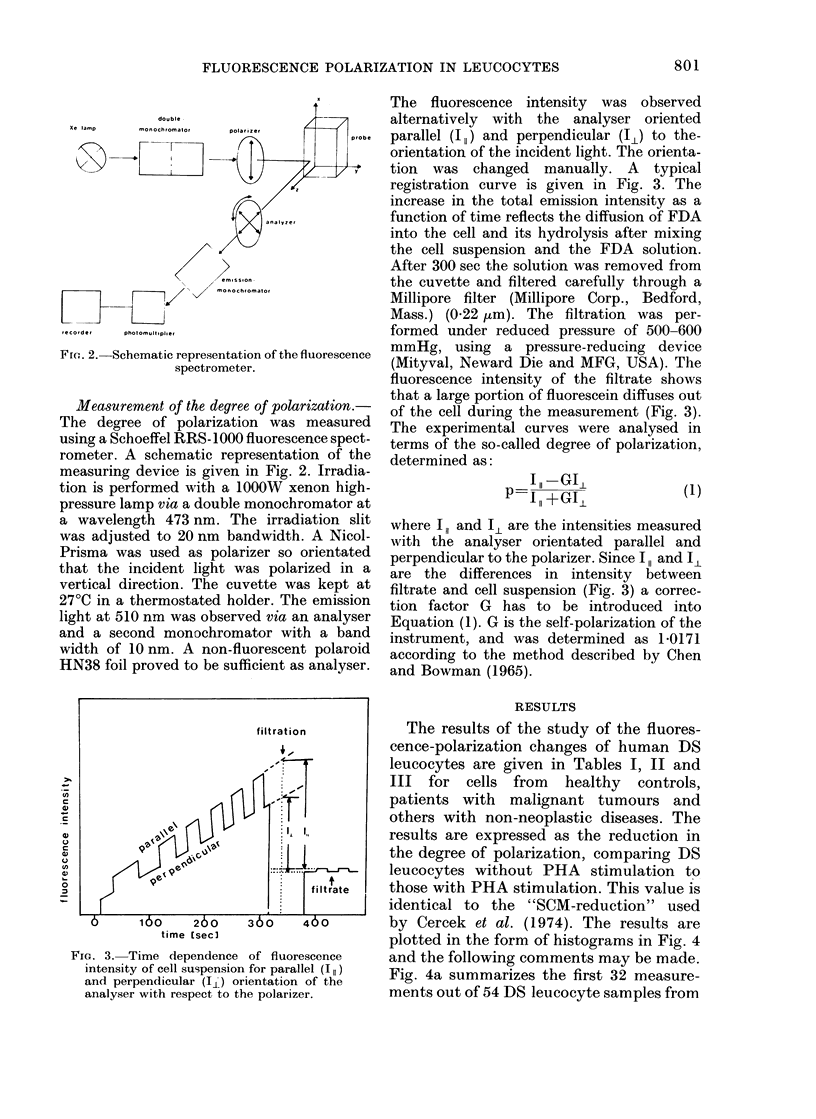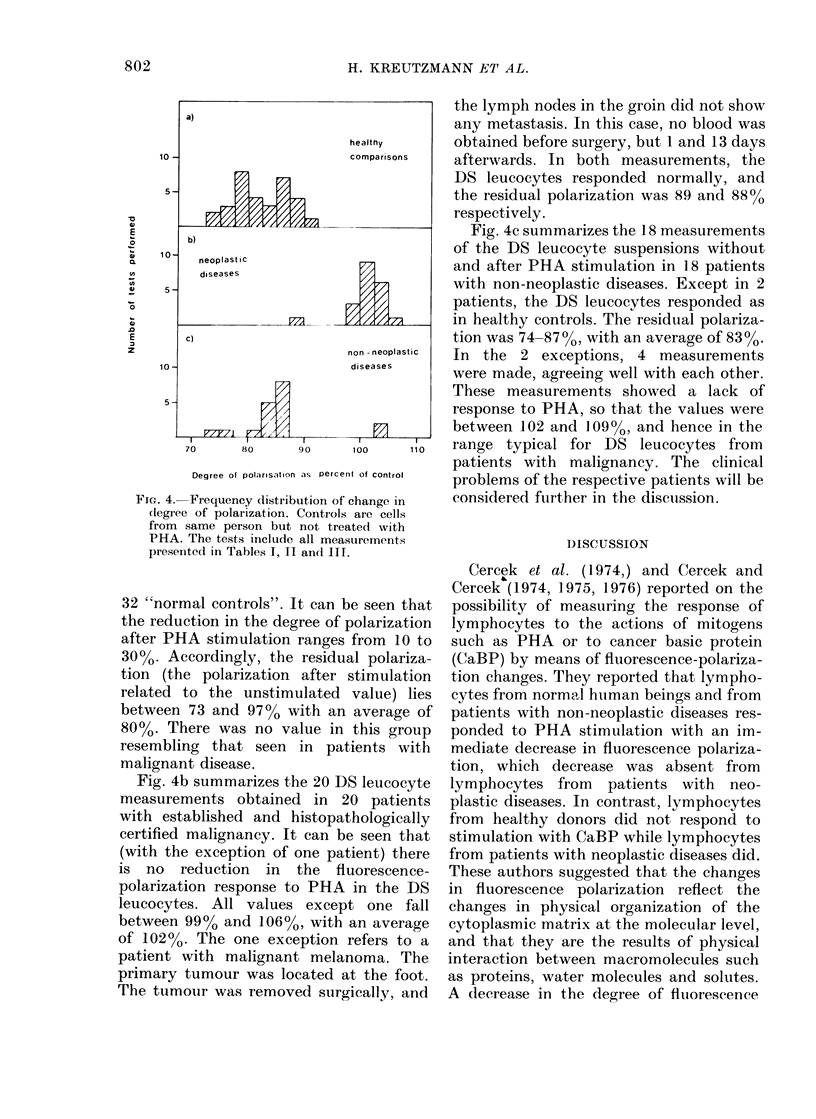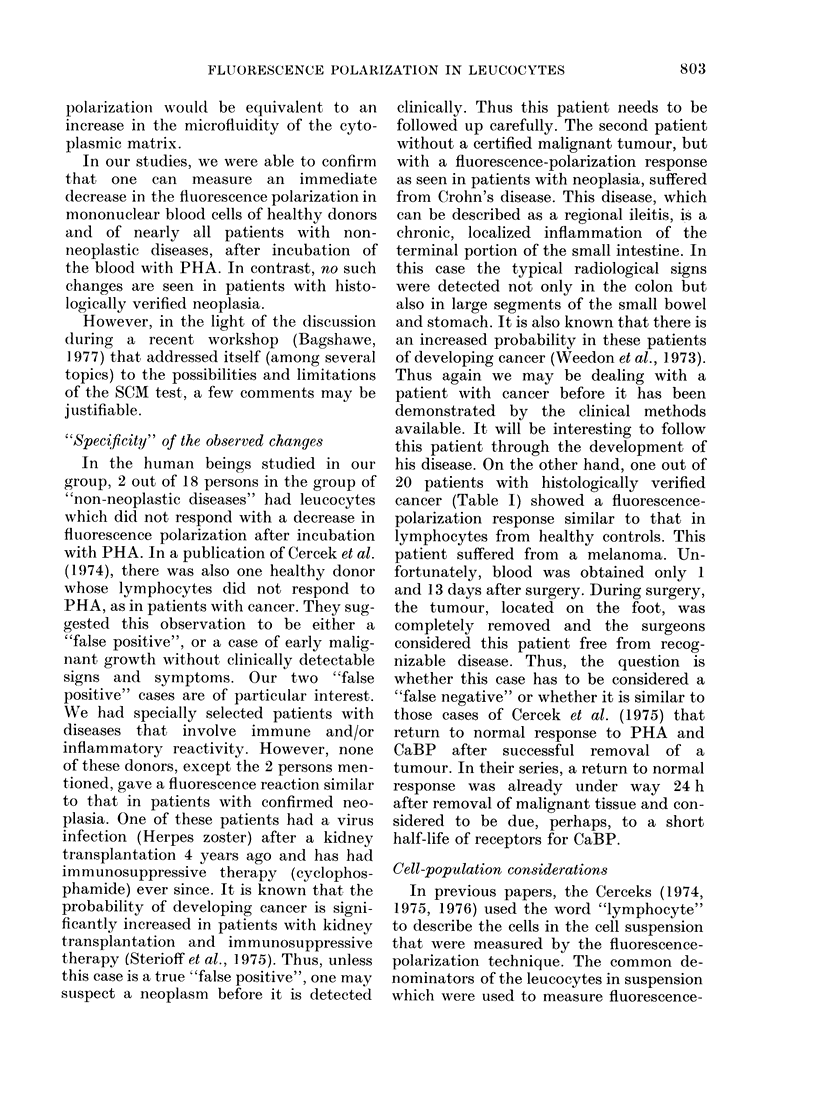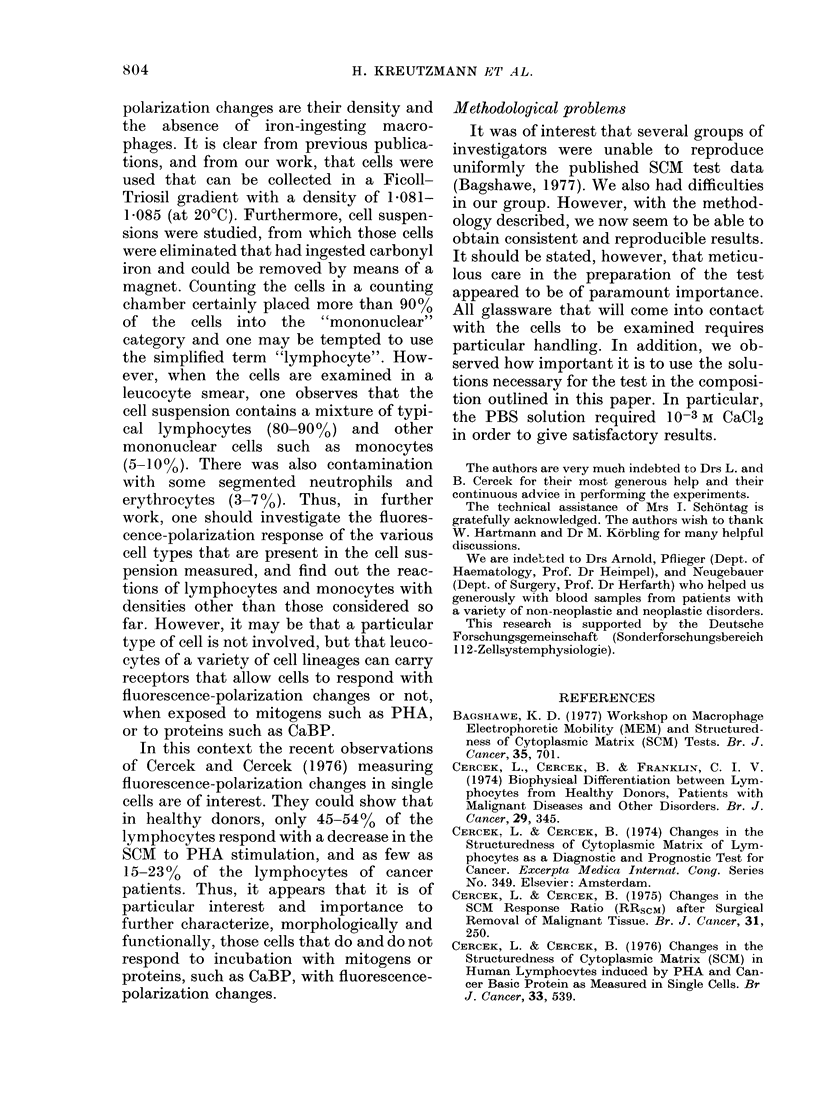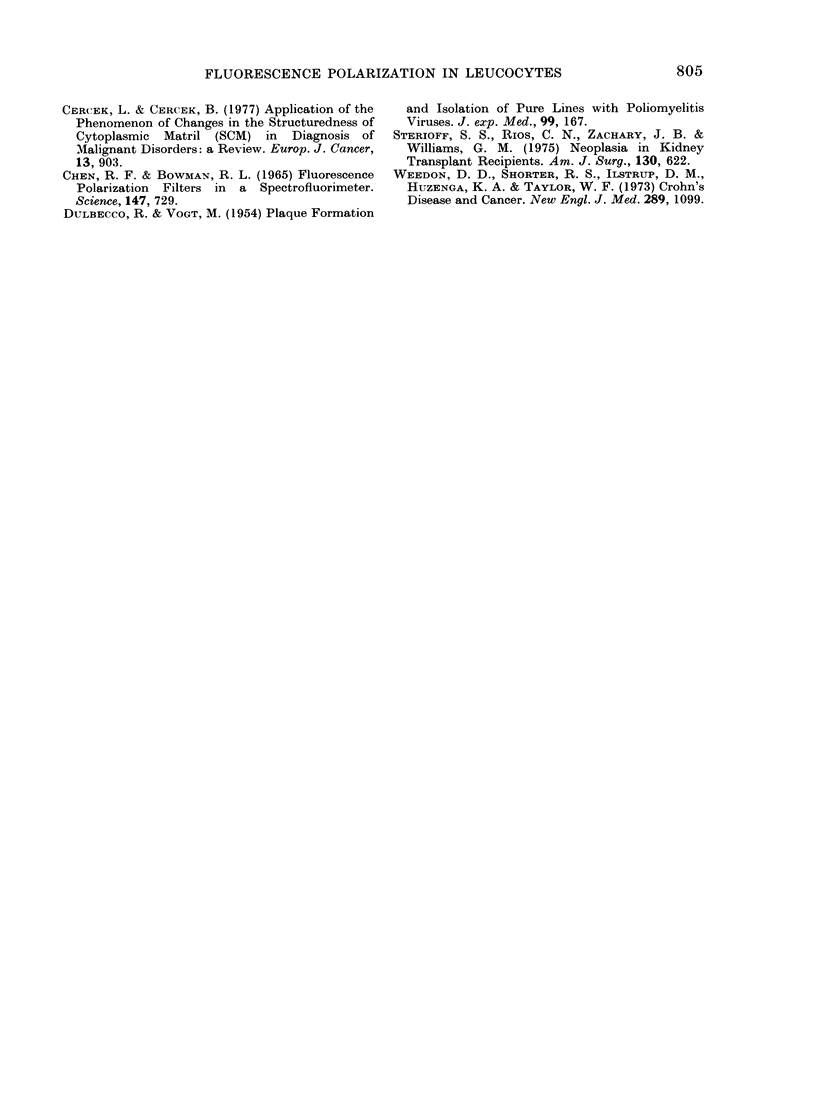# Fluorescence-polarization changes in mononuclear blood leucocytes after PHA incubation: differences in cells from patients with and without neoplasia.

**DOI:** 10.1038/bjc.1978.118

**Published:** 1978-05

**Authors:** H. Kreutzmann, T. M. Fliedner, H. J. Galla, E. Sackmann

## Abstract

**Images:**


*Br. J. Cancer* (1978) **37**, 797

### FLUORESCENCE-POLARIZATION CHANGES IN MONONUCLEAR BLOOD LEUCOCYTES AFTER PHA INCUBATION: DIFFERENCES IN CELLS FROM PATIENTS WITH AND WITHOUT NEOPLASIA

H. KREUTZMANN,* T. M. FLIEDNER,* H. J. GALLA† AND E. SACKMANN†

*From the *Department of Clinical Physiology and the †Department of Experimental Physics III, University of Ulm, Federal Republic of Germany*

Received 30 September 1977 Accepted 12 January 1978

**Summary.—In 32 healthy blood donors, 20 patients with histologically verified cancer and 18 patients with non-neoplastic diseases, the fluorescence polarization changes of fluorescein samples incorporated in mononuclear leucocytes were measured after incubation with PHA. The leucocytes of healthy persons, and 16/18 persons with non-neoplastic diseases, responded with a decrease in the degree of fluorescence polarization by about 20% from that in non-PHA-stimulated cells. In 19/20 patients with a variety of malignant tumours, the leucocytes did not respond to PHA stimulation with such a decrease. The exceptions among the patients with neoplastic and non-neoplastic diseases are considered, and may not be "false-negative" or "false-positive" respectively, but indicative of a particular situation in that disease. The biophysical mechanisms underlying the observed changes remain to be investigated.**

It has been the purpose of our studies to investigate the influence of phytohaemagglutinin (PHA) on the fluorescence polarization of fluorescein samples incorporated into mononuclear blood cells of normal human beings, of patients with histologically confirmed neoplasia, and of patients with non-neoplastic diseases. Previously, Cercek, Cercek and Franklin (1974) and Cercek and Cercek (1974, 1975, 1976) have published several reports which suggest that changes in fluorescence polarization after incubation of lymphocytes with PHA or with cancer basic proteins (CaBP) indicate specific changes of the "structuredness of the cytoplasmic matrix" (SCM). The "SCM-test", if it could be reproduced and confirmed, would represent a valuable tool in the early recognition of neoplasia, perhaps long before it could be detected by other means. It would also be useful to study the very early reactions of cells to foreign proteins on the structuredness of cytoplasm. In a recent workshop the potentialities of the SCM test were discussed and methodological problems raised (Bagshawe, 1977). Our studies show that there are fluorescence-polarization changes in mononuclear blood cells after their incubation with PHA which are absent when cells of the same density characteristics from patients with neoplasia are investigated. Reproducibility and reliability of the changes depend to a large extent on the methods used, and the care taken in the preparation of the cells for study. However, the mechanisms that lead to the observed changes are far from being clear, and require further investigation.

### MATERIALS AND METHODS

*Sources of mononuclear blood cells.*—Mononuclear blood cells were collected by drawing 20 ml of blood into heparinized polystyrene syringes from a total of 70 human beings.

Correspondence to: Professor Dr T. M. Fliedner, Abteilung für Klinische Physiologie der Universität Ulm, Oberer Eselsberg M24, D 7900 Ulm (Donau), Federal Republic of Germany.

The heparin concentration was 1000 iu/20 ml of blood (Liquemin 25000 from Hoffmann–La Roche). Thirty-two blood samples came from human volunteers without any known disease, who could be considered healthy by all accepted criteria. These blood samples were considered as from "normal controls" (Table I). Twenty-one blood samples were from patients with malignant tumours, the diagnosis of which was established by histopathological examination. The histopathological diagnoses are given in Table II. In 18 of the 21 samples, blood was taken before surgery. In the 3 exceptions (Nos. 3, 20a and 20b) the blood was removed after surgery (see Table). In Table III, the clinical diagnoses are given for those patients who did not suffer from known malignancy, but were admitted to the hospital for a variety of other reasons. The majority of these patients had infectious disease (bacterial, viral or fungal) or other inflammatory conditions.

TABLE I.—*Comparisons between Healthy Individuals*

| | | | Residual polarization as percentage of the unstimulated control value* | | |
|---|---|---|---|---|---|
| No. | Name | Age | 1st | 2nd | 3rd |
| 1 | HK | 30 | 88 | 73 | 81 |
| | | | 77 | 81 | 82 |
| 2 | GL | 31 | 87 | — | — |
| 3 | HA | 29 | 90 | — | — |
| 4 | WP | 31 | 89 | 88 | — |
| 5 | MP | 41 | 73 | 89 | — |
| 6 | KW | 27 | 85 | 90 | — |
| 7 | H-JG | 28 | 87 | 86 | — |
| 8 | USt | 28 | 88 | 87 | — |
| 9 | HM | 32 | 78 | 87 | — |
| 10 | MN | 35 | 89 | — | — |
| 11 | WS | 29 | 77 | — | — |
| 12 | KK | 28 | 82 | 73 | 78 |
| 13 | LOe | 27 | 87 | — | — |
| 14 | GF | 52 | 91 | — | — |
| 15 | HB | 25 | 78 | — | — |
| 16 | FR | 30 | 81 | — | — |
| 17 | HO | 35 | 79 | — | — |
| 18 | AG | 28 | 82 | 81 | — |
| 19 | ISch | 27 | 80 | 83 | — |
| 20 | EJ | 26 | 77 | — | — |
| 21 | NH | 29 | 74 | — | — |
| 22 | BK | 30 | 79 | — | — |
| 23 | HS | 27 | 82 | — | — |
| 24 | ES | 37 | 88 | 84 | — |
| 25 | AK | 30 | 87 | 77 | 85 |
| 26 | MS | 28 | 78 | — | — |
| 27 | WF | 40 | 85 | 75 | — |
| 28 | HJB | 36 | 87 | 85 | — |
| 29 | WK | 26 | 87 | — | — |
| 30 | GS | 25 | 85 | 86 | — |
| 31 | WG | 25 | 78 | — | — |
| 32 | DS | 26 | 80 | — | — |

* 1st, 2nd and 3rd measurements were carried out on different days.

The age of the healthy comparisons is lower than that of the patients with neoplasia because it was felt that the younger group is less likely to contain persons with undetected neoplasia that could distort the pattern. The group of volunteers comprises healthy associates of our research group and of other laboratories of our institutes. Only the first measurements were used for Fig. 4.

TABLE II.—*Patients with Malignant Diseases*

| No. | Name | Age | Diagnosis | Residual polarization as percentage of the unstimulated control value |
|---|---|---|---|---|
| 1 | VG | 77 | Adenoca rectum without LN metastases | 102 |
| 2 | ES | 60 | Adenoca colon | 102 |
| 3 | EU | 69 | Ca gall bladder with metastases to the liver after surgical removal | 104 |
| 4 | MR | 59 | Ca rectum | 101 |
| 5 | VP | 80 | Ca stomach | 99 |
| 6 | AK | 43 | Ca sigma | 101 |
| 7 | RB | 45 | Ca breast | 102 |
| 8 | AP | 54 | Ca breast | 104 |
| 9 | MH | 52 | Ca breast | 103 |
| 10 | BC | 77 | Ca stomach | 101 |
| 11 | RR | 78 | Ca stomach | 100 |
| 12 | EB | 54 | Ca pancreas | 106 |
| 13 | ER | 79 | Adeno ca rectum | 105 |
| 14 | US | 39 | Ca breast | 102 |
| 15 | EL | 79 | Lymphoma of stomach | 102 |
| 16 | RS | 67 | Ca pancreas | 103 |
| 17 | HS | 71 | Ca rectum | 99 |
| 18 | JS | 69 | Ca pancreas | 102 |
| 19 | IJ | 28 | Hodgkin's disease | 103 |
| 20 | FC | 50 | Melanoma | |
| a | | | 1 day } after surgical | 89 |
| b | | | 13 days } removal | 88 |

The blood was sampled and the cell suspensions were prepared by a physician. The measurements were all performed by a physicist not knowing the medical condition of the donor.

*Preparation of glassware.*—Pilot studies had indicated the need to prepare with utmost care all materials that came in contact with the cells to be studied. This included the special cleaning and sterilization of glass pipettes, test tubes, cuvettes, beakers, filtration tubes etc. The following procedure, as in Cercek and Cercek (1977), was applied. All the glassware was first immersed for at least 12 h in a 4% Extran(®) solution (Extran

TABLE III.—*Patients with Various Non-malignant Diseases*

| No. | Age | Diagnosis | Residual polarization as percentage of the unstimulated control value |
|---|---|---|---|
| 1 | 19 | Acute appendicitis | 86 |
| 2 | 44 | Fibroadenoma of breast | 74 |
| 3 | 65 | Polyp of colon | 87 |
| 4 | 24 | Rhinitis allergica | 85 |
| 5 | 68 | Tuberculosis | 86 |
| 6 | 64 | Tuberculosis | 87 |
| 7 | 46 | Cirrhosis of liver | 76 |
| 8 | 56 | Actinomycosis | 85 |
| 9 | 42 | Pneumonia | 86 |
| 10 | 30 | Herpes zoster after | 102* |
| | | kidney transplantation | 104 |
| 11 | 63 | Tuberculosis | 85 |
| 12 | 50 | Pneumonia | 84 |
| 13 | 24 | Crohn's disease and | 109* |
| | | polyposis of intestine | 105 |
| 14 | 20 | Pneumonia | 87 |
| 15 | 70 | Pneumonia | 86 |
| 16 | 50 | Pneumonia | 82 |
| 17 | 28 | Abscess of lung | 87 |
| 18 | 33 | Hepatitis | 85 |

* The two measurements in Nos. 10 and 13 were carried out on different days.

Solution, E. Merck, Darmstadt). This glassware was washed ×10 with hot water and then immersed for 8–12 h in chromic sulphuric acid. The glassware was then washed ×10 in hot water, immersed 4–6 h in double-distilled water, washed ×10 in double-distilled water, dried and sterilized in hot air (110°C).

*Preparation of solutions.*—The gradient solution used to separate mononuclear blood cells from the blood consisted of 24 parts of a 9% Ficoll (Pharmacia AB, Stockholm) solution and 10 parts of a 35·7% Triosil 440 (Nyegaard & Co., A.S. Oslo) solution. The Ficoll solution was passed through a millipore filter (0·22 $\mu$m) to sterilize it. It was important that the density of the gradient was reproducibly kept at 1·081–1·085 (at 20°C). If the gradient solution is to be kept for several days, it should be contained in a dark bottle at 4°C, since it is sensitive to light. Iron for removing phagocytic cells was the "Iron powder 99·5% ex iron carbonyl" commercially available (Koch-Light Laboratories Ltd).

The phosphate-buffered saline (PBS) used to wash the mononuclear blood cells and to obtain an osmolality of 330 mosm had the following composition per litre solution (Dulbecco and Vogt, 1954): 0·2 g KCl, 0·2 g $KH_2PO_4$, 2·91 g $Na_2$ $HPO_4$, $12H_2O$, 9·5 g NaCl, 6 ml of a stock solution (2·658 g/100 ml) $CaCl_2$, $2H_2O$, 5 ml of a stock solution (2·00 g/100 ml) $MgCl_2$. $6H_2O$. (all salts from E. Merck, Darmstadt). The PBS solution was sterilized by sterile filtration through a filter with a pore diameter of 0·22 $\mu$m.

The phytohaemagglutinin (PHA) solution was prepared as follows: Reagent Grade PHA (Wellcome Ltd) powder (45 mg) was dissolved in 5 ml distilled water. Aliquots of this solution were diluted ×5, from which 0·1 ml was added to the suspension of mononuclear blood leucocytes before study (see below).

The fluorescein diacetate solution (FDA Riedel-deHaen AG, Seelze-Hannover) was prepared as follows: 50 mg FDA was dissolved in 5 ml of glacial acetic acid. This solution has to be as pure as possible and should be kept at room temperature. 0·01 ml of this FDA stock solution was added to 100 ml PBS to which 0·37 g of $Na_2HPO_4$. $12H_2O$ were added. 25 ml of this FDA–PBS is then added to 75 ml of PBS. This final FDA–PBS solution should have an osmolality of 330 mosm and a pH of 7·4.

*Sampling and processing of blood.*—20 ml of blood was collected into a plastic syringe rinsed with heparin. In the laboratory, this blood was transferred into 2 glass screw-top vials, each containing 0·1 g of iron powder. The bottles were rotated (20–30 revs/min for 30 min) at 37°C. Thereafter, the bottles are placed over a magnet at a temperature of 20°C. Within about 10 min, the blood is free from iron-phagocytosing cells. Then the supernatant is removed and pipetted on to the Ficoll–Triosil gradient at 20°C in 16 mm-diameter glass tubes. Subsequently the gradient is subjected to centrifugation at 550 *g* (at the expected interphase) for 20 min.

One or two layers of cells then become visible. The upper layer or, if only one layer is present, the upper part is removed. Thus, the cells to be examined have in common a particular density which allows them to accumulate in this layer. These cells are therefore termed "density-specified leucocytes" or DS leucocytes. In the publications of Cercek *et al.* (1974) these cells are referred to as "SCM-responding lymphocytes". An aliquot of the cell suspension is removed to prepare smears for morphological examination (Fig. 1).

The remaining cell suspension is washed twice in 0·9% NaCl and once in PBS (see

FIG. 1.—Leucocyte concentrate smear of a cell suspension used for measurement after separation on the Ficoll–Triosil gradient. Upper, ×800; lower, ×1728.

above). The cells are then counted, and a cell suspension prepared with a concentration of not more than $6 \times 10^6$ DS leucocytes.

*Cell incubation with PHA.*—Aliquots of 1 ml of the cell suspension were incubated with 0·1 ml of PHA solution prepared as described above for 45–60 min at 37°C.

*Fluorescent probe.*—Fluorescein diacetate (FDA) is a non-fluorescent but fluorogenic substrate. It is transformed to fluorescein by enzymatic hydrolysis. It is this special feature of FDA which allows one to perform measurements in cell suspension. The fluorescence spectrum of fluorescein inside the cell is a typical broad emission spectrum with an emission maximum at 515 nm similar to that of fluorescein in solution. According to the suggestion of Cercek and Cercek (1977), the polarization measurements were performed at 510 nm.

FIG. 2.—Schematic representation of the fluorescence spectrometer.

*Measurement of the degree of polarization.*—The degree of polarization was measured using a Schoeffel RRS-1000 fluorescence spectrometer. A schematic representation of the measuring device is given in Fig. 2. Irradiation is performed with a 1000W xenon high-pressure lamp *via* a double monochromator at a wavelength 473 nm. The irradiation slit was adjusted to 20 nm bandwidth. A Nicol-Prisma was used as polarizer so orientated that the incident light was polarized in a vertical direction. The cuvette was kept at 27°C in a thermostated holder. The emission light at 510 nm was observed *via* an analyser and a second monochromator with a band width of 10 nm. A non-fluorescent polaroid HN38 foil proved to be sufficient as analyser.

FIG. 3.—Time dependence of fluorescence intensity of cell suspension for parallel ($I_{\parallel}$) and perpendicular ($I_{\perp}$) orientation of the analyser with respect to the polarizer.

The fluorescence intensity was observed alternatively with the analyser oriented parallel ($I_{\parallel}$) and perpendicular ($I_{\perp}$) to the orientation of the incident light. The orientation was changed manually. A typical registration curve is given in Fig. 3. The increase in the total emission intensity as a function of time reflects the diffusion of FDA into the cell and its hydrolysis after mixing the cell suspension and the FDA solution. After 300 sec the solution was removed from the cuvette and filtered carefully through a Millipore filter (Millipore Corp., Bedford, Mass.) (0·22 $\mu$m). The filtration was performed under reduced pressure of 500–600 mmHg, using a pressure-reducing device (Mityval, Neward Die and MFG, USA). The fluorescence intensity of the filtrate shows that a large portion of fluorescein diffuses out of the cell during the measurement (Fig. 3). The experimental curves were analysed in terms of the so-called degree of polarization, determined as:

$$p = \frac{I_{\parallel} - GI_{\perp}}{I_{\parallel} + GI_{\perp}} \qquad (1)$$

where $I_{\parallel}$ and $I_{\perp}$ are the intensities measured with the analyser orientated parallel and perpendicular to the polarizer. Since $I_{\parallel}$ and $I_{\perp}$ are the differences in intensity between filtrate and cell suspension (Fig. 3) a correction factor G has to be introduced into Equation (1). G is the self-polarization of the instrument, and was determined as 1·0171 according to the method described by Chen and Bowman (1965).

### RESULTS

The results of the study of the fluorescence-polarization changes of human DS leucocytes are given in Tables I, II and III for cells from healthy controls, patients with malignant tumours and others with non-neoplastic diseases. The results are expressed as the reduction in the degree of polarization, comparing DS leucocytes without PHA stimulation to those with PHA stimulation. This value is identical to the "SCM-reduction" used by Cercek *et al.* (1974). The results are plotted in the form of histograms in Fig. 4 and the following comments may be made. Fig. 4a summarizes the first 32 measurements out of 54 DS leucocyte samples from

FIG. 4.—Frequency distribution of change in degree of polarization. Controls are cells from same person but not treated with PHA. The tests include all measurements presented in Tables I, II and III.

32 "normal controls". It can be seen that the reduction in the degree of polarization after PHA stimulation ranges from 10 to 30%. Accordingly, the residual polarization (the polarization after stimulation related to the unstimulated value) lies between 73 and 97% with an average of 80%. There was no value in this group resembling that seen in patients with malignant disease.

Fig. 4b summarizes the 20 DS leucocyte measurements obtained in 20 patients with established and histopathologically certified malignancy. It can be seen that (with the exception of one patient) there is no reduction in the fluorescence-polarization response to PHA in the DS leucocytes. All values except one fall between 99% and 106%, with an average of 102%. The one exception refers to a patient with malignant melanoma. The primary tumour was located at the foot. The tumour was removed surgically, and the lymph nodes in the groin did not show any metastasis. In this case, no blood was obtained before surgery, but 1 and 13 days afterwards. In both measurements, the DS leucocytes responded normally, and the residual polarization was 89 and 88% respectively.

Fig. 4c summarizes the 18 measurements of the DS leucocyte suspensions without and after PHA stimulation in 18 patients with non-neoplastic diseases. Except in 2 patients, the DS leucocytes responded as in healthy controls. The residual polarization was 74–87%, with an average of 83%. In the 2 exceptions, 4 measurements were made, agreeing well with each other. These measurements showed a lack of response to PHA, so that the values were between 102 and 109%, and hence in the range typical for DS leucocytes from patients with malignancy. The clinical problems of the respective patients will be considered further in the discussion.

### DISCUSSION

Cercek *et al.* (1974,) and Cercek and Cercek (1974, 1975, 1976) reported on the possibility of measuring the response of lymphocytes to the actions of mitogens such as PHA or to cancer basic protein (CaBP) by means of fluorescence-polarization changes. They reported that lymphocytes from normal human beings and from patients with non-neoplastic diseases responded to PHA stimulation with an immediate decrease in fluorescence polarization, which decrease was absent from lymphocytes from patients with neoplastic diseases. In contrast, lymphocytes from healthy donors did not respond to stimulation with CaBP while lymphocytes from patients with neoplastic diseases did. These authors suggested that the changes in fluorescence polarization reflect the changes in physical organization of the cytoplasmic matrix at the molecular level, and that they are the results of physical interaction between macromolecules such as proteins, water molecules and solutes. A decrease in the degree of fluorescence polarization would be equivalent to an increase in the microfluidity of the cytoplasmic matrix.

In our studies, we were able to confirm that one can measure an immediate decrease in the fluorescence polarization in mononuclear blood cells of healthy donors and of nearly all patients with non-neoplastic diseases, after incubation of the blood with PHA. In contrast, *no* such changes are seen in patients with histologically verified neoplasia.

However, in the light of the discussion during a recent workshop (Bagshawe, 1977) that addressed itself (among several topics) to the possibilities and limitations of the SCM test, a few comments may be justifiable.

*"Specificity" of the observed changes*

In the human beings studied in our group, 2 out of 18 persons in the group of "non-neoplastic diseases" had leucocytes which did not respond with a decrease in fluorescence polarization after incubation with PHA. In a publication of Cercek *et al.* (1974), there was also one healthy donor whose lymphocytes did not respond to PHA, as in patients with cancer. They suggested this observation to be either a "false positive", or a case of early malignant growth without clinically detectable signs and symptoms. Our two "false positive" cases are of particular interest. We had specially selected patients with diseases that involve immune and/or inflammatory reactivity. However, none of these donors, except the 2 persons mentioned, gave a fluorescence reaction similar to that in patients with confirmed neoplasia. One of these patients had a virus infection (Herpes zoster) after a kidney transplantation 4 years ago and has had immunosuppressive therapy (cyclophosphamide) ever since. It is known that the probability of developing cancer is significantly increased in patients with kidney transplantation and immunosuppressive therapy (Sterioff *et al.*, 1975). Thus, unless this case is a true "false positive", one may suspect a neoplasm before it is detected clinically. Thus this patient needs to be followed up carefully. The second patient without a certified malignant tumour, but with a fluorescence-polarization response as seen in patients with neoplasia, suffered from Crohn's disease. This disease, which can be described as a regional ileitis, is a chronic, localized inflammation of the terminal portion of the small intestine. In this case the typical radiological signs were detected not only in the colon but also in large segments of the small bowel and stomach. It is also known that there is an increased probability in these patients of developing cancer (Weedon *et al.*, 1973). Thus again we may be dealing with a patient with cancer before it has been demonstrated by the clinical methods available. It will be interesting to follow this patient through the development of his disease. On the other hand, one out of 20 patients with histologically verified cancer (Table I) showed a fluorescence-polarization response similar to that in lymphocytes from healthy controls. This patient suffered from a melanoma. Unfortunately, blood was obtained only 1 and 13 days after surgery. During surgery, the tumour, located on the foot, was completely removed and the surgeons considered this patient free from recognizable disease. Thus, the question is whether this case has to be considered a "false negative" or whether it is similar to those cases of Cercek *et al.* (1975) that return to normal response to PHA and CaBP after successful removal of a tumour. In their series, a return to normal response was already under way 24 h after removal of malignant tissue and considered to be due, perhaps, to a short half-life of receptors for CaBP.

*Cell-population considerations*

In previous papers, the Cerceks (1974, 1975, 1976) used the word "lymphocyte" to describe the cells in the cell suspension that were measured by the fluorescence-polarization technique. The common denominators of the leucocytes in suspension which were used to measure fluorescencepolarization changes are their density and the absence of iron-ingesting macrophages. It is clear from previous publications, and from our work, that cells were used that can be collected in a Ficoll–Triosil gradient with a density of 1·081–1·085 (at 20°C). Furthermore, cell suspensions were studied, from which those cells were eliminated that had ingested carbonyl iron and could be removed by means of a magnet. Counting the cells in a counting chamber certainly placed more than 90% of the cells into the "mononuclear" category and one may be tempted to use the simplified term "lymphocyte". However, when the cells are examined in a leucocyte smear, one observes that the cell suspension contains a mixture of typical lymphocytes (80–90%) and other mononuclear cells such as monocytes (5–10%). There was also contamination with some segmented neutrophils and erythrocytes (3–7%). Thus, in further work, one should investigate the fluorescence-polarization response of the various cell types that are present in the cell suspension measured, and find out the reactions of lymphocytes and monocytes with densities other than those considered so far. However, it may be that a particular type of cell is not involved, but that leucocytes of a variety of cell lineages can carry receptors that allow cells to respond with fluorescence-polarization changes or not, when exposed to mitogens such as PHA, or to proteins such as CaBP.

In this context the recent observations of Cercek and Cercek (1976) measuring fluorescence-polarization changes in single cells are of interest. They could show that in healthy donors, only 45–54% of the lymphocytes respond with a decrease in the SCM to PHA stimulation, and as few as 15–23% of the lymphocytes of cancer patients. Thus, it appears that it is of particular interest and importance to further characterize, morphologically and functionally, those cells that do and do not respond to incubation with mitogens or proteins, such as CaBP, with fluorescence-polarization changes.

### *Methodological problems*

It was of interest that several groups of investigators were unable to reproduce uniformly the published SCM test data (Bagshawe, 1977). We also had difficulties in our group. However, with the methodology described, we now seem to be able to obtain consistent and reproducible results. It should be stated, however, that meticulous care in the preparation of the test appeared to be of paramount importance. All glassware that will come into contact with the cells to be examined requires particular handling. In addition, we observed how important it is to use the solutions necessary for the test in the composition outlined in this paper. In particular, the PBS solution required $10^{-3}$ M $CaCl_2$ in order to give satisfactory results.

The authors are very much indebted to Drs L. and B. Cercek for their most generous help and their continuous advice in performing the experiments.

The technical assistance of Mrs I. Schöntag is gratefully acknowledged. The authors wish to thank W. Hartmann and Dr M. Körbling for many helpful discussions.

We are indebted to Drs Arnold, Pflieger (Dept. of Haematology, Prof. Dr Heimpel), and Neugebauer (Dept. of Surgery, Prof. Dr Herfarth) who helped us generously with blood samples from patients with a variety of non-neoplastic and neoplastic disorders.

This research is supported by the Deutsche Forschungsgemeinschaft (Sonderforschungsbereich 112-Zellsystemphysiologie).

### REFERENCES

Bagshawe, K. D. (1977) Workshop on Macrophage Electrophoretic Mobility (MEM) and Structuredness of Cytoplasmic Matrix (SCM) Tests. *Br. J. Cancer*, **35**, 701.

Cercek, L., Cercek, B. & Franklin, C. I. V. (1974) Biophysical Differentiation between Lymphocytes from Healthy Donors, Patients with Malignant Diseases and Other Disorders. *Br. J. Cancer*, **29**, 345.

Cercek, L. & Cercek, B. (1974) Changes in the Structuredness of Cytoplasmic Matrix of Lymphocytes as a Diagnostic and Prognostic Test for Cancer. *Excerpta Medica Internat. Cong.* Series No. 349. Elsevier: Amsterdam.

Cercek, L. & Cercek, B. (1975) Changes in the SCM Response Ratio ($RR_{SCM}$) after Surgical Removal of Malignant Tissue. *Br. J. Cancer*, **31**, 250.

Cercek, L. & Cercek, B. (1976) Changes in the Structuredness of Cytoplasmic Matrix (SCM) in Human Lymphocytes induced by PHA and Cancer Basic Protein as Measured in Single Cells. *Br J. Cancer*, **33**, 539.

CERCEK, L. & CERCEK, B. (1977) Application of the Phenomenon of Changes in the Structuredness of Cytoplasmic Matril (SCM) in Diagnosis of Malignant Disorders: a Review. *Europ. J. Cancer*, **13**, 903.

CHEN, R. F. & BOWMAN, R. L. (1965) Fluorescence Polarization Filters in a Spectrofluorimeter. *Science*, **147**, 729.

DULBECCO, R. & VOGT, M. (1954) Plaque Formation and Isolation of Pure Lines with Poliomyelitis Viruses. *J. exp. Med.*, **99**, 167.

STERIOFF, S. S., RIOS, C. N., ZACHARY, J. B. & Williams, G. M. (1975) Neoplasia in Kidney Transplant Recipients. *Am. J. Surg.*, **130**, 622.

WEEDON, D. D., SHORTER, R. S., ILSTRUP, D. M., HUZENGA, K. A. & TAYLOR, W. F. (1973) Crohn's Disease and Cancer. *New Engl. J. Med.* **289**, 1099.